# Design and rationale of the efficacy of spinal cord stimulation in patients with refractory angina pectoris (SCRAP) trial

**DOI:** 10.1002/clc.24016

**Published:** 2023-04-04

**Authors:** F. E. Vervaat, A. van der Gaag, C. Smetsers, P. C. Barneveld, M. van't Veer, K. Teeuwen, H. van Suijlekom, L. Dekker, I. F. Wijnbergen

**Affiliations:** ^1^ Department of Cardiology Catharina Hospital Eindhoven The Netherlands; ^2^ Department of Anesthesiology Catharina Hospital Eindhoven The Netherlands; ^3^ Department of Nuclear Medicine Jeroen Bosch Hospital ‘s‐Hertogenbosch The Netherlands

**Keywords:** refractory angina pectoris, spinal cord stimulation, trial design

## Abstract

**Background:**

The use of spinal cord stimulation (SCS) in patients with refractory angina pectoris (RAP) is still under debate. Studies up to date have shown a positive effect with an improvement in quality of life. However, no double blinded randomized controlled trials have been performed.

**Hypothesis & Methods:**

The objective of this trial is to investigate if high density SCS leads to a significant reduction in the amount of myocardial ischemia in patients with RAP. Eligible patients must meet the criteria for RAP, have proven ischemia and a positive transcutaneous electrical nerve stimulator treadmill test. Patients who meet the inclusion criteria will receive an implanted spinal cord stimulator. Patients receive 6 months of high density SCS and 6 months of no stimulation using a cross‐over design. The order of the treatment options is determined using randomization. The primary endpoint is the effect of SCS measured by the change in percentage of myocardial ischemia using myocardial perfusion positron emission tomography scan. Key secondary endpoints are patient related outcome measures, major cardiac adverse events and safety endpoints. The follow‐up period is 1 year for the primary and key secondary endpoints.

**Results:**

The SCRAP trial began enrollment on December 21, 2021 and is set to complete the primary assessments in June 2025. To date, January 2, 2023, 18 patients have been enrolled in the study and 3 patients have completed the 1‐year follow‐up.

**Conclusions:**

The SCRAP trial is an investigator‐initiated, single‐center, double‐blind, placebo‐controlled, and cross‐over randomized controlled trial investigating the efficacy of SCS in patients with RAP. (ClinicalTrials. gov Identifier: NCT04915157)

## Graphical abstract

Overview of the design of the “Efficacy of Spinal Cord Stimulation in Patients with Refractory Angina Pectoris (SCRAP) trial.” AP = angina pectoris, TENS = transcutaneous electrical nerve stimulation, PET = positron emission tomography, SCS = spinal cord stimulation, SAQ = seattle angina questionnaire, RAND‐36 = RAND 36‐item health survey, 6‐MWT = 6‐min walking test, NRS = numeric rating scale, CCS = Canadian cardiovascular society.

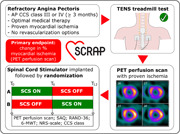

## 1 INTRODUCTION

Coronary artery disease (CAD) is one of the most prevalent diseases worldwide. Due to advancements in treatment options mortality and morbidity rates in this patient population have steadily declined. However, up to 20% of patients with stable CAD have persisting angina symptoms.^1^ It is estimated that 5%–10% of patients with stable CAD who remain symptomatic despite optimal treatment have “refractory angina pectoris” (RAP).^2^, ^3^ RAP was defined in 2002 by Mannheimer et al as a chronic condition (>3 months) characterized by diffuse coronary artery disease in the presence of proven ischemia, which is not amenable to a combination of medical therapy, angioplasty or coronary bypass surgery.^2^ In absolute numbers up to 1.8 million people in the United States (US) have RAP with 50 000–100 000 new cases each year in the United States and 30 000–50 000 new cases each year in Europe.^4^ Patients with RAP have a severely limited quality of life due to the daily, persisting angina symptoms with its inherent physical limitations.

Spinal cord stimulation (SCS) is a last resort treatment option for this patient population in which no other treatment options are currently available. SCS is a device with a lead located in the higher thoracic epidural space and an implantable pulse generator (IPG) in the abdomen or buttock that provides stimulation at the level of the myocardial afferent sympathetic neurons (C7–Th1). The potential beneficial effects of SCS are based on four possible mechanisms: reduction of myocardial oxygen demand, reduction of pain perception, and a decrease in sympathetic tone and an improvement in the coronary microcirculatory blood flow.^5^, ^6^

The majority of research into the effect of SCS on RAP has been observational in nature. To date three placebo‐controlled randomized controlled trials have been performed and one study compared SCS with usual care.^6^, ^7^, ^8^, ^9^ In the three placebo‐controlled studies various settings of spinal cord stimulation (normal, subthreshold, and sham) have been compared with differing results.^7^, ^8^, ^9^ Reduction in the number and severity of angina symptoms was proven in all studies, but it is not clear if there is a placebo effect as suggested by the study of Zipes et al.^9^ In this study two modes of stimulation; high—conventional use of spinal cord stimulation, and low—spinal cord stimulation during 1 min each day, were compared with no significant differences between the two groups after 6 months of treatment.^9^ Whilst the two other studies comparing different levels of spinal cord stimulation did find significant differences between the groups.^7^, ^8^ These conflicting results make interpretation difficult, especially taking into account that the studies were underpowered and had slow patient inclusion rates which led to early termination of the studies. Despite the limitations of these individual studies two systematic reviews confirm that there is a significant improvement in exercise duration, Canadian Cardiovascular Society (CCS) class, Visual Analog Scale (VAS) score, daily angina episodes, angina frequency, daily nitrate consumption, disease perception and treatment satisfaction in RAP patients with SCS.^10^, ^11^

Although the exact working mechanism of SCS in RAP has not been elucidated studies have repeatedly shown an improvement in the quality of life and a reduction in angina symptoms.^7^, ^8^, ^9^, ^11^, ^12^, ^13^ It is unclear if SCS leads to reduction in the amount of myocardial ischemia. One study by Diedrichs et al. determined the effect of SCS based on the amount of myocardial ischemia using Methoxyisobutylisonitrile single photon emission computed tomography (MIBI‐SPECT).^14^ All patients were treated with SCS and MIBI‐SPECT was performed at baseline, 3 and 12 months. After 12 months, there was a significant reduction in the amount of myocardial ischemia that was not seen at 3 months. Because the reduction of myocardial ischemia was only seen at 12 months, the authors concluded that this effect might not have been a direct effect of SCS, but rather due to better coronary collateralization created by enhanced physical activity. Thus, the question remains whether SCS actually reduces the amount of myocardial ischemia.

The recently published European Society of Cardiology (ESC) guideline “Chronic Coronary Syndromes” describes varying levels of evidence with regard to treatment options in patients with RAP. The ESC guideline concludes that SCS may be considered (Class of recommendation IIB; level of evidence B).^15^ It summarizes that “Larger RCTs are required to define the role of each treatment modality for specific subgroups, to decrease nonresponder rates and ascertain benefit beyond potential placebo effects,” confirming the need for additional studies.^15^

The type of SCS traditionally used in patients with RAP is conventional stimulation. With this type of stimulation the patient will feel paresthesia at the target area. For this study a paresthesia free form of stimulation, high density (HD) stimulation, will be applied. Based on previous experience it is a viable treatment modality, it allows adequate blinding of patients because no paresthesia are felt and the patient can be fully informed about the fact that the spinal cord stimulator will be turned off for a period of 6 months.^16^ This is in contrast to previous studies in which blinding of patients has been problematic due to the paresthesia felt by the patient.^6^, ^7^, ^8^, ^9^

The aim of this investigator‐initiated, single‐center, placebo‐controlled, double‐blind, cross‐over, randomized controlled trial is to determine if high density spinal cord stimulation, a paresthesia free form of stimulation, leads to a significant reduction in the amount of myocardial ischemia in patients with RAP. The trial has been registered at ClinicalTrials.gov with NCT identifier NCT04915157.

## 2 METHODS

### 2.1 Study design

The primary objective of this study is to determine the effect of SCS in patients with RAP on the percentage of myocardial ischemia measured using a myocardial perfusion positron emission tomography (PET) scan. Patients with RAP referred to our hospital (Catharina hospital, Eindhoven, the Netherlands) will be screened for eligibility to participate in the study. The inclusion criteria must be met, meaning stable angina pectoris CCS class III or IV during at least the previous 3 months, a coronary angiogram (CAG) performed within the last 12 months with significant CAD not suitable for intervention, optimal antianginal medication and proven ischemia using MIBI‐SPECT, perfusion magnetic resonance imaging (MRI), myocardial perfusion PET scan or a fractional flow reserve (FFR) positive lesion (<0.80) with no intervention options. Tables 1 and 2 give a complete overview of the inclusion and exclusion criteria. To determine if patients meet the afore mentioned criteria the referral letter and CAG images will be evaluated by one of the interventional cardiologists (two in case of doubt).

**Table 1:** Inclusion criteria.

Refractory Angina Pectoris: ○ Stable angina pectoris CCS class III or IV, with a minimum of five episodes of angina pectoris over the course of 1 week, during a minimum period of 3 months before screening; ○ CAG performed within the last 12 months showing significant coronary artery disease defined as at least one coronary artery stenosis of >75% or 50%–75% with proven ischemia (see below), not suitable for revascularization. Confirmed by one (or two in case of doubt) interventional cardiologist based on CAG images; ○ Optimal antianginal medication. Patients should at least use the maximal tolerable dose of a beta‐blocker, calcium channel blocker and short‐ and/or long‐acting nitrate. If the patient does not use one of these groups of medication the reason (side‐effects) should be clear.
Proven ischemia: ○ MIBI‐SPECT: SSS of at least 1, in combination with SDS of at least 1 (1–4 mildischemia, >4 moderate to severe ischemia). ○ Fractional flow reserve: <0.80, with no intervention options (determined by interventional cardiologist). ○ MRI perfusion: ≥1 segment of subendocardial hypoperfusion during stress perfusion, not present at rest and no matching fibrosis (using 16 segment AHA heart model). ○ Myocardial perfusion PET: Semi‐quantitative measurement: SSS of at least 1, in combination with SDS of at least 1 (1–4 mild ischemia, >4 moderate to severe ischemia). Quantitative measurement: reduced myocardial perfusion reserve.
No revascularization (PCI and/or CABG) performed between ischemia testing and study inclusion.
Age >18 years.

Abbreviations: CABG, coronary artery bypass grafting; CAG, coronary angiogram; MIBI‐SPECT, methoxyisobutylisonitrile single photon emission computed tomography; MRI, magnetic resonance imaging; PCI, percutaneous coronary intervention; PET, positron emission tomography; SDS, summed difference score; SSS, summed stress score.

**Table 2:** Exclusion criteria.

Acute coronary syndrome during the 3‐month period before screening.
Life expectancy less than 12 months.
Inability to perform a 6‐min walking test.
Inability to give informed consent.
No proven ischemia (see Table 1 Inclusion criteria).
Spinal cord disease which could prevent correct positioning of the lead in the epidural space; to be determined by the anesthesiologist performing the implantation.
Anticoagulation therapy that cannot be stopped/bridged before spinal cord stimulator implantation.
Inadequate paresthesia coverage, during implantation, of the thoracic region where angina complaints are localized.
Pregnancy.
Mild Cognitive Impairment or dementia.
Concomitant symptomatic valvular heart disease including severe aortic stenosis and/or regurgitation, severe mitral valve stenosis and/or regurgitation or severe tricuspid valve regurgitation.

#### 2.1.1 Standardized TENS treadmill test

Eligible patients will be invited to the outpatient clinic to perform a standardized transcutaneous electrical nerve stimulation (TENS) treadmill test. The standardized TENS treadmill test is used as a screening tool, as advised by Mannheimer et al in the ESC report, to determine if the patient is eligible for an implanted spinal cord stimulator.^2^ The standardized TENS treadmill test is used to induce angina pectoris and determine what the effect is of TENS on the time to resolution of angina pectoris. For the standardized TENS treadmill test two adhesive electrodes are applied to the chest and connected to a battery‐operated TENS (eco 2, Schwa‐medico Netherlands BV). The TENS settings are programmed with a pulse width of 200µs and a frequency of 2 Hz. The patient is asked to start walking on a treadmill (Enraf‐Nonius BV) with an initial speed of 3.5 km/h. The speed is increased every 30 s with 0.5 km/h to a maximum of 5.5 km/h. If angina pectoris has not been induced the inclination angle is increased with 1% every 30 s. Once angina pectoris is induced the treadmill will be stopped, followed by switching on the TENS with an amplitude of 40 mA. A timer is started and the patient will be asked to comment when the angina pectoris is no longer present.

Before the standardized TENS treadmill test the patient will fill out the TENS questionnaire (Appendix 1). The answer to question 2 of the TENS questionnaire—usual time until symptom resolution without use of short‐acting nitrates, will be compared to the time it takes for the symptoms to disappear after initiation of TENS. There are three possible test outcomes:

(a) Positive: equal to or more than 50% reduction in the time from onset of angina pectoris until resolution of angina pectoris after TENS initiation, compared to the “normal” time it takes for angina pectoris to resolve.
(b) Dubious: less than 50% reduction in the time from onset of angina pectoris until resolution of angina pectoris after TENS initiation, compared to the ‘normal’ time it takes for angina pectoris to resolve.
(c) Negative: no reduction or increase in the time from onset of angina pectoris until resolution of angina pectoris after TENS initiation, compared to the “normal” time it takes for angina pectoris to resolve.

**Figure 1:**
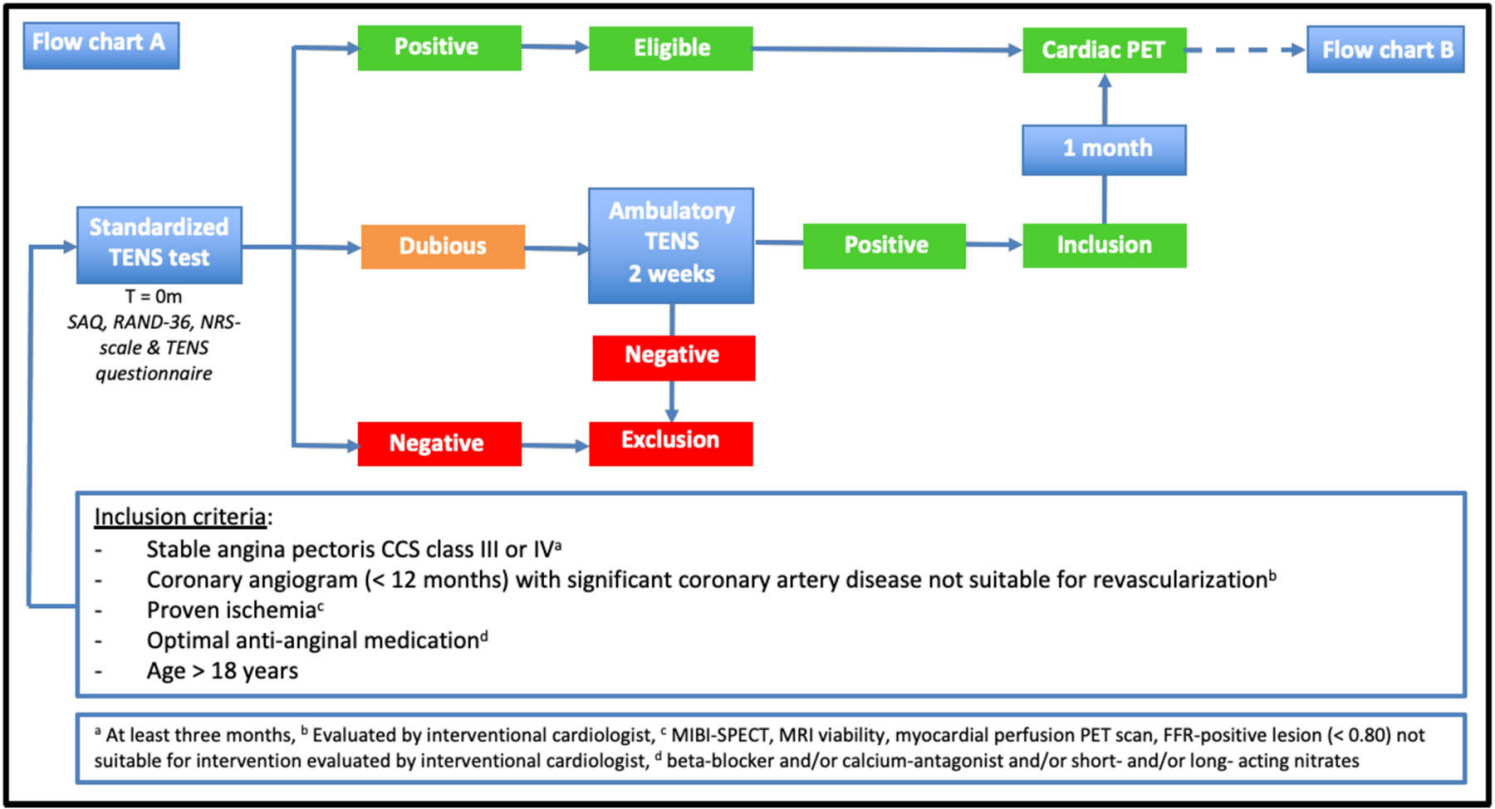
Flowchart of the screening process for inclusion into the SCRAP trial; patients eligible for participation in the SCRAP trial. A standardized TENS treadmill test is performed. Patients with a dubious standardized TENS treadmill test will be included if they have a positive result after 2 weeks of ambulatory TENS. Patients with a positive standardized TENS treadmill test will undergo a cardiac PET. CCS, canadian cardiovascular society; FFR, fractional flow reserve; MIBI‐SPECT, methoxyisobutylisonitrile single photon emission computed tomography; MRI,  magnetic resonance imaging; NRS, numeric rating scale; PET, positron emission tomography; RAND‐36, RAND 36‐item Health Survey; SAQ, seattle angina questionnaire; TENS, transcutaneous electrical nerve stimulation.

Patients with a positive standardized TENS treadmill test are eligible for participation in the study and a myocardial perfusion PET scan is performed to determine the amount of myocardial ischemia present. Patients with a negative standardized TENS treadmill test are excluded from the study. Patients with a dubious standardized TENS treadmill test will receive TENS treatment in an ambulatory setting for a duration of 2 weeks. After this period the effect of TENS will be re‐evaluated by the same team to determine whether the effect was positive or negative (a dubious test result is not possible). If the effect is positive the patient is eligible and a myocardial perfusion PET scan will be performed 1 month after termination of TENS treatment (washout period to prevent possible lingering effects of the TENS treatment) (Figure 1).

**Figure 2:**
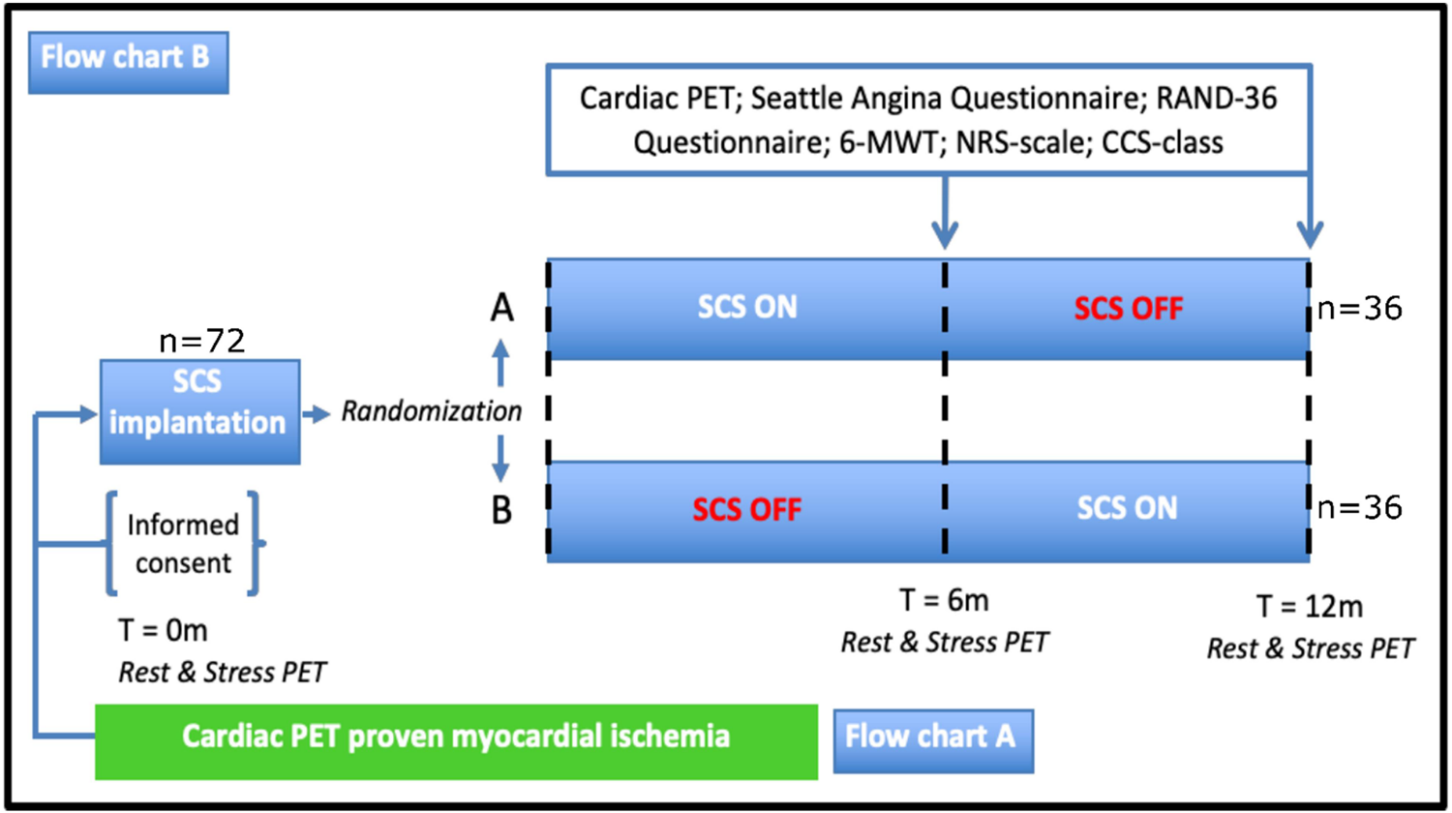
Flowchart of randomization process and study design; after informed consent is given a SCS is implanted. Patients are randomized into arm A or B with 6 months of spinal cord stimulation and 6 months of no stimulation or vice versa. At 6 and 12 months measurements (including PET, 6‐MWT, SAQ, NRS, CCS, and RAND‐36) are performed. The study period is 12 months. 6‐MWT, 6 min walking test; NRS, numeric rat; PET, positron emission tomography, RAND‐36, RAND 36‐item Health Survey; SCS, spinal cord stimulator.

If myocardial ischemia is proven, with myocardial perfusion PET scanning, the patient is included. All participating patients will receive an implanted spinal cord stimulator. The total duration of the study is 12 months during which patients will receive 6 months of HD SCS, a paresthesia free form of SCS, and 6 months of no stimulation using a cross‐over design. To determine the order of the treatment options randomization using the online program Research Manager will be performed (Figure 2). The randomization is done by the study nurse. The study nurse will be the only person of research team that knows to which arm the patient has been randomized. This same study nurse is also responsible for inputting the correct programming of the SCS based on the randomization. The patient and the remaining members of the research team are blinded to the randomization process. During the study period of one year the patient will have use of the patient programmer. To ensure blinding of the patient remains intact, during the “SCS OFF” or placebo period, a non‐existent second lead will be programmed as being active, whilst leading to no actual SCS. This enables the use of the patient programmer during the “SCS OFF” or placebo period and also keeps the blinding of the patient intact.

#### 2.1.2 Spinal cord stimulator implantation

The spinal cord stimulator system consists of an implantable pulse generator (IPG) (PrimeADVANCED™ SureScan® MRI Medtronic), a single lead (Vectris Compact™ SureScan MRI Medtronic) and a patient programmer (myStim Patient Programmer & ADVANCED™ Antenna Medtronic). The spinal cord stimulator implantation will take place in an operating room in accordance with national guidelines concerning aseptic preparations. During the procedure the patient is asked to lie in a prone position to increase the thoracic kyphosis. Moderate sedation is applied using a combination of propofol and remifentanil, secondary field blocks and wound infiltration with local anesthetics will be used to provide analgesia. Sedation levels and vital signs are controlled and monitored by an experienced nurse. A Tuohy needle is used to enter the epidural space at the level of thoracic vertebrae (Th) 7–8. Entrance into the epidural space is gained using loss of resistance and confirmed by advancing a guide wire through the Tuohy needle under fluoroscopic guidance. Next the guide wire is removed and the lead is advanced into the epidural space with the cephalad tip positioned paramedian to the left side at the level of C7‐Th1 using fluoroscopic guidance. To determine if the position of the lead is correct sedation is temporarily lifted and using an external programmer (Intellis Wireless External NeuroStimulator (WENS), Samsung Programsystem and Communicator) stimulations are given. The aim is to achieve a paresthesia coverage of the target area (left side of the chest) of at least 80% and subtle modifications of the lead position are performed to reach the optimal coverage based on feedback from the patient during test stimulation. When the optimal position of the lead has been determined the lead is fixated at the thoracodorsal fascia, subcutaneously tunneled and connected to the IPG pocket. The usual subcutaneous position of the IPG is the left buttock. After the implantation has been completed the patient is monitored on the ward and an X‐ray is made to confirm the position of the lead. Depending on the randomization the correct settings will be input by the unblinded study nurse (Appendix 2 gives a detailed description of the spinal cord stimulator settings). In general, the patient will be discharged from the hospital on the same day with instructions on the use of the patient programmer and what to do if complications and/or questions arise.

### 2.2 Endpoints

#### 2.2.1 Primary endpoint objective

The primary objective of this study is the effect of SCS in patients with RAP measured by the change in the percentage of myocardial ischemia (% of the left ventricular myocardium) using myocardial perfusion PET scan at the end of the six‐month period of treatment with SCS compared with baseline.

#### 2.2.2 Secondary endpoint objectives

An overview of the secondary objectives is given in Table 3. The secondary objectives are patient related outcome measures (PROMs), major adverse cardiac events (MACE) and safety endpoints. MACE consists of the following components; (i) number of hospital admissions due to an acute coronary syndrome (ACS), (ii) undergoing revascularization (PCI and/or CABG), (iii) number of presentations at the emergency room due to angina pectoris and, (iv) cardiovascular mortality. ACS is defined in accordance with the universal definition of myocardial infarction as a high‐sensitivity troponin T with at least one value above the 99th percentile of the upper reference limit with at least one of the following; (1) symptoms of myocardial ischemia, (2) new ischemic electrocardiographic changes, (3) development of pathological Q waves, (4) imaging evidence of loss of viable myocardium or new regional wall motion abnormality or, (5) intracoronary thrombus detected on angiography or autopsy.^17^ The clinical endpoints will be investigator reported. The Seattle Angina questionnaire (SAQ; Appendix 3), RAND 36‐item Health Survey (RAND‐36; Appendix 4) questionnaire, Numeric Rating Scale (NRS‐scale; Appendix 5), CCS class, 6‐min walking test (6‐MWT), and review of patient files will be used to obtain the secondary objectives (Table 3).

**Table 3:** Secondary objectives.

Changes in absolute quantification of myocardial blood flow using myocardial perfusion PET scan including myocardial blood flow & myocardial flow reserve regionally and globally.
Patient related outcome measures: ○ Patient condition using 6‐minute walking test; ○ Frequency of angina pectoris using the SAQ; ○ Severity of angina pectoris using the Numeric Rating Scale; ○ Grading of angina pectoris using the Canadian Cardiovascular Society class; ○ Quality of life using the RAND 36‐item Health Survey; ○ Frequency of short‐acting nitrate use using the SAQ.
Major Adverse Cardiac Events: ○ Number of hospital admissions due to acute coronary syndrome; ○ Revascularization (CABG and/or PCI); ○ Number of presentations at the emergency room due to angina pectoris; ○ Cardiovascular mortality.
Safety endpoints; ○ Number of device infections (lead and/or battery); ○ Number of device dislocations (lead and/or battery); ○ Number of lead fractures/breakages; ○ Number of lead failures; ○ Number of battery end of life.

Abbreviations: CABG, coronary artery bypass grafting; PCI, percutaneous coronary intervention; PET, positron emission tomography; SAQ, Seattle angina questionnaire.

### 2.3 Follow‐up

At baseline the SAQ, RAND‐36, NRS‐scale, and CCS class will be completed when the patient is seen for the initial standardized TENS treadmill test. The baseline 6‐MWT will be performed after the baseline myocardial perfusion PET scan has shown myocardial ischemia. After spinal cord stimulator implantation the follow‐up appointments will take place at the outpatient clinic of the department of anesthesiology. Patients will be seen at the outpatient clinic one week (to check the surgical wounds), 1, 3, 6 months, and 1 year after initial spinal cord stimulator implantation.

At 6 months the myocardial perfusion PET scan will be repeated before the 6‐month outpatient clinic appointment. The SAQ, RAND‐36, NRS‐scale, CCS class, and 6‐MWT will be completed when the patient is seen for the 6‐month outpatient clinic visit. After confirmation that all data are complete by a member of the research team (excluding the study nurse), the patient has an appointment with the study nurse. At this appointment the study nurse will change the programming according to the randomization, from “SCS ON” to “SCS OFF” or vice versa. The programming of the spinal cord stimulator is performed in the same manner and by the same study nurse as was done at baseline ensuring that the blinding of the patient and the rest of the research team remains intact. At 12 months the myocardial perfusion PET scan will be repeated before the final, 12‐month outpatient clinic appointment. Additionally the SAQ, RAND‐36, NRS‐scale, CCS class, and 6‐MWT will be completed when the patient is seen for the final outpatient clinic appointment by a member of the research team in the same fashion as the 6 month appointment. At this final appointment, after confirmation of complete data, the spinal cord stimulator settings will be set to conventional spinal cord stimulation by the study nurse in the same manner as the 6 month appointment. In addition the patient will be asked during which period of the study, the first or second 6 months, it was thought that the spinal cord stimulator was turned on.

For the duration of the study patients are asked to contact our center to inform us if visits to the emergency room, admissions to hospital and/or the undergoing of a PCI and/or CABG have occurred. If complications arise related to the implanted spinal cord stimulator (i.e., signs of infection, battery dislocation, lead dislocation, and/or lead fracture) patients will be asked to contact the outpatient clinic of the department of anesthesiology to determine the appropriate course of action.

After completion of the study patients will be invited to the outpatient clinic each year for a total duration of 5 years for long‐term follow‐up. At each outpatient clinic appointment patients will be asked to complete the SAQ, RAND‐36, NRS‐scale, CCS class, and 6‐MWT.

### 2.4 Sample size calculation

Currently, no data with regard to the percentage of myocardial ischemia (as % of the left ventricular myocardium) present in patients with RAP using myocardial perfusion PET scan as imaging modality are available. The data available on this subject is with the imaging modality MIBI‐SPECT. The percentage of myocardial ischemia (as % of the left ventricular myocardium) present in patients with refractory angina pectoris using MIBI‐SPECT ranges from 6.12% (standard deviation of 3.7)^18^ up to 14.01%.^19^ Combining this data, it translates into an average percentage of myocardial ischemia of 10% with a standard deviation of 7.

Using these clinical assumptions (average percentage of myocardial ischemia of 10% and a standard deviation of 7) and the typical assumptions (paired, two‐tailed *T* test, *α* .05 and power .80) a sample size of 32 patients per arm (total 64 patients) could detect a relative reduction in percentage of myocardial ischemia of 25%. Assuming a 10% patient drop‐out in the analysis, this means a total of 72 patients should be included in the study.

Due to the study design using cross‐over, it is theoretically possible that a carry‐over and/or period effect could occur. We will apply the two‐stage approach as proposed by Grizzle et al to assess the carry‐over and period effect.^20^ To assess the carry‐over effect we will first determine the summed effect over the two periods and compare these between the two groups (A and B). In the same way we will assess the period effect by determining the summed effect over the two groups and compare these between the periods. When analyses show that the carry‐over effect as well as the period effect are negligible, we will use a paired, two‐tailed Students’ *T* test for the main outcome parameter. If a significant carry‐over effect is found, only data from the first period will be analyzed. Due to the long study period of 6 months before performing the PET scan after cross‐over has taken place, the assumption is that there will not be a carry‐over effect.

### 2.5 Organization and ethical concerns

This is a single‐center, investigator‐initiated study. The study protocol has been approved by the local ethics committee. All patients will provide written informed consent before study participation. All investigators will adhere to Good Clinical Practice and to the ethical principles of the Declaration of Helsinki.

An independent Data Safety Monitoring Board (DSMB) consisting of an interventional cardiologist, anesthesiologist and statistician has been established for this study to oversee safety. A blinded interim analysis will be performed after 24 patients have been included and additional interim analysis will be performed when deemed necessary by the DSMB. The DSMB will have access to the unblinded study data (including patient characteristics, invasive procedure details, primary and secondary efficacy endpoints, safety endpoints and relevant serious adverse events) and can give the following recommendations: (i) proceed with the trial according to protocol, (ii) modify the study protocol or study procedures (including, but not limited to, changes in inclusion/exclusion criteria, frequency of safety monitoring, alterations in study procedures and follow‐up period for the purposes of safety), and (iii) terminate inclusion of new patients into the trial.

## 3 RESULTS

Study enrollment started on December 21, 2021 and the estimated completion of the primary assessments is June 2025. To date, January 2, 2023, a total of 18 patients have been enrolled in the study. Three patients have completed the 1‐year follow‐up. The baseline characteristics of the patients currently enrolled are shown in Supporting Information: Table 1.

## 4 DISCUSSION

The SCRAP trial will be conducted in a large specialized hospital in the Netherlands (Catharina Hospital, Eindhoven). In the Netherlands, the “National Health Care Institute” evaluates certain aspects of our health care system to determine if a treatment modality is effective and should be part of insured health care. In November 2019, the National Health Care Institute evaluated the treatment modality neuromodulation in patients with chronic pain, including patients with RAP. The report concluded that, based on the available literature (June 2018), spinal cord stimulation should not be considered as an effective treatment modality in patients with refractory angina pectoris and should therefore no longer be part of insured health care in the Netherlands.^21^ An important note in the report was the lack of randomized controlled trials in patients with RAP and spinal cord stimulation. However previous results from our center show that spinal cord stimulation leads to significantly improved quality of life with significant reduction in angina pectoris episodes and less use of short‐acting nitrates.^13^ Additionally in the ESC guideline “chronic coronary syndromes” varying levels of evidence with regard to treatment options for patients with RAP have been described.^15^ The guideline recommendation for use of SCS in RAP is Class IIB; level of evidence B—may be considered. With the conclusion that additional research is necessary to determine the exact role of the various treatment modalities for RAP. Taking all the afore mentioned points together it can be concluded that the strength of the studies performed up to date has been insufficient to definitively show a positive effect of SCS in patients with RAP. And with the report of the National Health Care Institute it is currently not possible for treating cardiologists in the Netherlands to refer patients with RAP to specialized centers, such as the Catharina Hospital, for SCS treatment, emphasizing the urgent need for studies such as the SCRAP trial to clarify the role of SCS in patients with RAP.

The decision to use changes in myocardial ischemia, measured with myocardial perfusion PET scanning, as primary endpoint is based on the afore mentioned report of the National Health Care Institute. An important comment in the report was that evidence of reduction in myocardial ischemia is currently missing, which led to the formulation of the chosen primary endpoint.^21^ Taking into consideration the importance of patient related outcome measures (PROMs) key secondary endpoints are PROM outcomes.

The choice of 6 months SCS treatment is based on previous research that has shown that changes can be seen in myocardial blood flow (using PET) after 3–6 weeks of SCS therapy.^22^, ^23^ Whilst also taking into consideration that changes in myocardial ischemia (using MIBI) were not yet seen after 3 months of SCS therapy in the study by Diedrichs et al.^14^

An important concern voiced in previous literature is the risk of adverse events related to the spinal cord stimulator implantation in general. Adverse events range from lead dislocation to device related infections with a reported incidence of up to 40%.^3^, ^24^ Spinal cord stimulation is used in a variety of chronic pain syndromes such as RAP and failed back surgery syndrome (FBSS). One review reported on adverse events specifically related to patients with RAP and SCS. This review described 1% of patients with an infection (1 out of 104 patients) and 7.8% of patients with lead migration or fracture (10 out of 128 patients).^25^ As is true for all interventional therapies operator experience will lead to a reduction of the number of adverse events over time. Our center has been implanting spinal cord stimulators for the indication RAP since 2009 and a recent observational study carried out in our center showed a low percentage of adverse events over a follow‐up period of one year (2.3%; 2 out of 87 patients),^13^ showing that the risk of adverse events is low in patients with RAP receiving a spinal cord stimulator. The risk of a complication related to the spinal cord stimulator implantation has been taken into consideration in the design of the SCRAP trial in two ways. First, the drop‐out rate of 10% was imbedded into the sample size calculation and secondly in the SCRAP trial adverse events related to the spinal cord stimulator implantation have been added as safety end points (Table 3) and will be reported at the end of the study period.

## 5 LIMITATIONS

There are a number of limitations with regard to the SCRAP trial. One limitation is the difference in myocardial ischemia on which the sample size calculation is based. The power of the study is based on a relative reduction in myocardial ischemia of 25% after treatment with SCS, in absolute numbers a reduction from 10% to 7.5% of myocardial ischemia. Whilst this is a relatively modest difference, it is deemed clinically relevant. Patients with more than 10% myocardial ischemia have a worse prognosis in comparison to patients with less than 10% myocardial ischemia,^15^ making the chosen difference clinically relevant. A second limitation of the study is the single‐center design and the reason for this choice is twofold. Firstly the number of patients meeting the selection criteria is expected to be low. Second, the implantation of spinal cord stimulators should be limited to specialized centers to reduce the risk of device‐related complications and the chosen center for the SCRAP trial is such a specialized center.^26^, ^27^

## 6 CONCLUSION

The SCRAP trial is an investigator‐initiated, single‐center, double‐blind, placebo‐controlled, cross‐over randomized controlled trial evaluating the efficacy of spinal cord stimulation on the amount of myocardial ischemia in patients with refractory angina pectoris.

## CONFLICT OF INTEREST STATEMENT

The authors declare no conflict of interest.

## Supporting information

Supporting information.

Supporting information.

Supporting information.

Supporting information.

Supporting information.

Supporting information.

## Data Availability

The data that support the findings of this study are available from the corresponding author, FEV, upon reasonable request.
