# Supplementary material for: Design and rationale of the efficacy of spinal cord stimulation in patients with refractory angina pectoris (SCRAP) trial
Source: Clin Cardiol. 2023 Apr 4;46(6):689–97. doi: 10.1002/clc.24016 (PMC10270247; doi:10.1002/clc.24016)
Supplement: Supplementary file 1 — Supporting information. [file CLC-46-689-s004.docx]

**Appendix 1**

1. After how many metres walking at a normal pace do you experience an episode of chest pain (angina pectoris) or shortness of breath, forcing you to stop this activity (completely or temporarily)?

…… meters.

2. When you experience an episode of chest pain (angina pectoris) or shortness of breath, at rest or during exercise, after how much time (seconds or minutes) do the symptoms disappear if you DON’T use additional medication (spray or tablet short-acting nitroglycerine)?

…… seconds/minutes.

3. When you experience an episode of chest pain (angina pectoris) or shortness of breath, at rest or during exercise, after how much time (seconds or minutes) do the symptoms disappear if you DO use additional medication (spray or tablet short-acting nitroglycerine)?

…… seconds/minutes.

TENS Questionnaire (original version in Dutch).
