# Supplementary material for: Design and rationale of the efficacy of spinal cord stimulation in patients with refractory angina pectoris (SCRAP) trial
Source: Clin Cardiol. 2023 Apr 4;46(6):689–97. doi: 10.1002/clc.24016 (PMC10270247; doi:10.1002/clc.24016)
Supplement: Supplementary file 2 — Supporting information. [file CLC-46-689-s006.docx]

**Appendix 2**

*Spinal cord stimulator settings*

After successful implantation of the spinal cord stimulator the programming will be performed by the study nurse. The programming procedure is the same in each patient. The patient will be in a supine position and using conventional stimulation the position of the lead will be checked by confirming the location (left chest region) where the paresthesia are felt by the patient. Next the spinal cord stimulator will be set to HD stimulation with the patient still in a supine position. For the HD stimulation settings the frequency will be 260Hz and the pulse width 450ms for all patients participating in the study. The only parameter of the HD stimulation settings that will vary for each patient is the amplitude (ranging from 0.5 up to 8mA). To determine the correct amplitude for the patient HD stimulation is applied with increasing amplitude until felt by the patient; this amplitude is defined as the 100% sensory threshold. The amplitude will be lowered to 70% of the sensory threshold to ensure the patient will not feel paresthesia. To ensure blinding during the study period the patient can only increase the amplitude up to 80% of the sensory threshold using the patient programmer. The next step is to test the HD stimulation in a standing position and also during coughing (leads to elevated pressure on the lead) at 70% of the sensory threshold increasing the amplitude up to 80% of the sensory threshold. If paresthesia are felt by the patient, the process will be repeated with a lower amplitude. Once it has been confirmed that the patient feels no paresthesia at 70% and 80% of the sensory threshold for the HD stimulation settings are finalized. Following finalization the study nurse will perform the randomization and will program the spinal cord stimulator to “SCS ON” – HD stimulation on the implanted lead – or “SCS OFF” – no stimulation by programming the non-existent second lead. At 6 months cross-over will take place and this afore mentioned process will be repeated by the same study nurse.
