# Supplementary material for: Design and rationale of the efficacy of spinal cord stimulation in patients with refractory angina pectoris (SCRAP) trial
Source: Clin Cardiol. 2023 Apr 4;46(6):689–97. doi: 10.1002/clc.24016 (PMC10270247; doi:10.1002/clc.24016)
Supplement: Supplementary file 3 — Supporting information. [file CLC-46-689-s002.docx]

**Appendix 3** – Seattle Angina Questionnaire

Spertus JA et al. Development and Evaluation of the Seattle Angina Questionnaire: A New Functional Status Measure for Coronary Artery Disease. J Am Coll Cardiol 1995;25:333-41
