# Supplementary material for: Design and rationale of the efficacy of spinal cord stimulation in patients with refractory angina pectoris (SCRAP) trial
Source: Clin Cardiol. 2023 Apr 4;46(6):689–97. doi: 10.1002/clc.24016 (PMC10270247; doi:10.1002/clc.24016)
Supplement: Supplementary file 4 — Supporting information. [file CLC-46-689-s005.docx]

**Appendix 4** – RAND 36-Item Health Survey

Source: [36-Item Short Form Survey Instrument (SF-36) | RAND](https://www.rand.org/health-care/surveys_tools/mos/36-item-short-form/survey-instrument.html)

**Choose one option for each questionnaire item.**

Bovenkant formulier

1. In general, would you say your health is:

 1 - Excellent

 2 - Very good

 3 - Good

 4 - Fair

 5 - Poor

2. **Compared to one year ago**, how would you rate your health in general **now**?

 1 - Much better now than one year ago

 2 - Somewhat better now than one year ago

 3 - About the same

 4 - Somewhat worse now than one year ago

 5 - Much worse now than one year ago

The following items are about activities you might do during a typical day. Does **your health now limit you** in these activities? If so, how much?

|  | Yes, limited a lot | Yes, limited a little | No, not limited at all |
| --- | --- | --- | --- |
| 3. **Vigorous activities**, such as running, lifting heavy objects, participating in strenuous sports |  1 |  2 |  3 |
| 4. **Moderate activities**, such as moving a table, pushing a vacuum cleaner, bowling, or playing golf |  1 |  2 |  3 |
| 5. Lifting or carrying groceries |  1 |  2 |  3 |
| 6. Climbing **several** flights of stairs |  1 |  2 |  3 |
| 7. Climbing **one** flight of stairs |  1 |  2 |  3 |
| 8. Bending, kneeling, or stooping |  1 |  2 |  3 |
| 9. Walking **more than a mile** |  1 |  2 |  3 |
| 10. Walking **several blocks** |  1 |  2 |  3 |
| 11. Walking **one block** |  1 |  2 |  3 |
| 12. Bathing or dressing yourself |  1 |  2 |  3 |

During the **past 4 weeks**, have you had any of the following problems with your work or other regular daily activities **as a result of your physical health**?

|  | Yes | No |
| --- | --- | --- |
| 13. Cut down the **amount of time** you spent on work or other activities |  1 |  2 |
| 14. **Accomplished less** than you would like |  1 |  2 |
| 15. Were limited in the **kind** of work or other activities |  1 |  2 |
| 16. Had **difficulty** performing the work or other activities (for example, it took extra effort) |  1 |  2 |

During the **past 4 weeks**, have you had any of the following problems with your work or other regular daily activities **as a result of any emotional problems** (such as feeling depressed or anxious)?

|  | Yes | No |
| --- | --- | --- |
| 17. Cut down the **amount of time** you spent on work or other activities |  1 |  2 |
| 18. **Accomplished less** than you would like |  1 |  2 |
| 19. Didn't do work or other activities as **carefully** as usual |  1 |  2 |

20. During the **past 4 weeks**, to what extent has your physical health or emotional problems interfered with your normal social activities with family, friends, neighbors, or groups?

 1 - Not at all

 2 - Slightly

 3 - Moderately

 4 - Quite a bit

 5 - Extremely

21. How much **bodily** pain have you had during the **past 4 weeks**?

 1 - None

 2 - Very mild

 3 - Mild

 4 - Moderate

 5 - Severe

 6 - Very severe

22. During the **past 4 weeks**, how much did **pain** interfere with your normal work (including both work outside the home and housework)?

 1 - Not at all

 2 - A little bit

 3 - Moderately

 4 - Quite a bit

 5 - Extremely

These questions are about how you feel and how things have been with you **during the past 4 weeks**. For each question, please give the one answer that comes closest to the way you have been feeling.

How much of the time during the **past 4 weeks**...

|  | All of the time | Most of the time | A good bit of the time | Some of the time | A little of the time | None of the time |
| --- | --- | --- | --- | --- | --- | --- |
| 23. Did you feel full of pep? |  1 |  2 |  3 |  4 |  5 |  6 |
| 24. Have you been a very nervous person? |  1 |  2 |  3 |  4 |  5 |  6 |
| 25. Have you felt so down in the dumps that nothing could cheer you up? |  1 |  2 |  3 |  4 |  5 |  6 |
| 26. Have you felt calm and peaceful? |  1 |  2 |  3 |  4 |  5 |  6 |
| 27. Did you have a lot of energy? |  1 |  2 |  3 |  4 |  5 |  6 |
| 28. Have you felt downhearted and blue? | 1 | 2 | 3 | 4 | 5 | 6 |
| 29. Did you feel worn out? | 1 | 2 | 3 | 4 | 5 | 6 |
| 30. Have you been a happy person? | 1 | 2 | 3 | 4 | 5 | 6 |
| 31. Did you feel tired? | 1 | 2 | 3 | 4 | 5 | 6 |

32. During the **past 4 weeks**, how much of the time has **your physical health or emotional problems** interfered with your social activities (like visiting with friends, relatives, etc.)?

 1 - All of the time

 2 - Most of the time

 3 - Some of the time

 4 - A little of the time

 5 - None of the time

How TRUE or FALSE is **each** of the following statements for you.

|  | Definitely true | Mostly true | Don't know | Mostly false | Definitely false |
| --- | --- | --- | --- | --- | --- |
| 33. I seem to get sick a little easier than other people | 1 | 2 | 3 | 4 | 5 |
| 34. I am as healthy as anybody I know | 1 | 2 | 3 | 4 | 5 |
| 35. I expect my health to get worse | 1 | 2 | 3 | 4 | 5 |
| 36. My health is excellent | 1 | 2 | 3 | 4 | 5 |

Onderkant formulier
