# Supplementary material for: Design and rationale of the efficacy of spinal cord stimulation in patients with refractory angina pectoris (SCRAP) trial
Source: Clin Cardiol. 2023 Apr 4;46(6):689–97. doi: 10.1002/clc.24016 (PMC10270247; doi:10.1002/clc.24016)
Supplement: Supplementary file 5 — Supporting information. [file CLC-46-689-s001.docx]

**Appendix 5** – Numeric Pain Rating Scale

**Numeric Pain Rating Scale**

*Select the number that best reflects the severity of your pain.*

How sever was your pain (on average) during the past week (7 days)?

0 1 2 3 4 5 6 7 8 9 10

**No pain Most severe**

**pain**
