# Supplementary material for: Design and rationale of the efficacy of spinal cord stimulation in patients with refractory angina pectoris (SCRAP) trial
Source: Clin Cardiol. 2023 Apr 4;46(6):689–97. doi: 10.1002/clc.24016 (PMC10270247; doi:10.1002/clc.24016)
Supplement: Supplementary file 6 — Supporting information. [file CLC-46-689-s003.docx]

**Supplementary figures/tables**

| Supplementary table 1: Patient characteristics at baseline | |
| --- | --- |
|  | n = 18 |
| *General* | |
| Age - yr | 66.4 ± 10.3 |
| Male – no. (%) | 18 (100) |
| *Cardiovascular risk factors* | |
| BMI – kg/m^2^ | 28.8 ± 4.06 |
| Smoking status – no. (%)   - Current tobacco user - Previous tobacco user | 1 (5.6)  12 (66.7) |
| Dyslipidemia – no (%) | 15 (83.3) |
| Diabetes mellitus – no (%) | 5 (27.8) |
| Family history of coronary artery disease – no (%) | 7 (38.9) |
| *Medical history* | |
| Previous myocardial infarction – no (%) | 11 (61.1) |
| Previous PCI – no (%) | 16 (88.9) |
| Previous CABG – no (%) | 12 (66.7) |
| Peripheral artery disease – no (%) | 4 (22.2) |
| Previous stroke – no (%) | 3 (16.7) |
| Internal cardiac device – no (%) | 2 (11.1) |
| LVEF - % | 57.1 ± 11.6 |
| Three vessel coronary artery disease – no (%) | 14 (77.8) |
| *Current medication* |  |
| Aspirin – no (%) | 14 (77.8) |
| P_2_Y_12_-inhibitor – no (%) | 8 (44.4) |
| Oral anticoagulants – no (%) | 4 (22.2) |
| Beta-blocker – no (%) | 12 (66.7)* |
| Calcium-antagonist – no (%) | 12 (66.7)* |
| RAAS-inhibitor – no (%) | 12 (66.7)** |
| Long-acting nitrates – no (%) | 16 (88.9)* |
| Short-acting nitrates – no (%) | 18 (100) |
| Lipid lowering therapy – no (%)   - Statins - Ezetimib - PCSK9 inhibitor - Gemfibrozil | 18 (100)  13 (72.2)  7 (38.9)  5 (27.8)  1 (5.6) |
| Ivabradine – no (%) | 2 (11.1) |
| Nicorandil – no (%) | 4 (22.2) |

Footnote: * remaining patients did not use medication due to side-effects. ** ACE-inhibitor or angiotensin receptor blocker. BMI = body mass index, PCI = percutaneous coronary intervention, CABG = coronary artery bypass grafting, LVEF = left ventricular ejection fraction, RAAS = Renin-angiotensin-aldosteron system.
